# Unusual intramuscular locations as a first presentation of hydatid cyst disease in children: a report of two cases

**DOI:** 10.1186/s12887-021-02843-5

**Published:** 2021-08-31

**Authors:** Ruba A. Khasawneh, Ziyad M. Mohaidat, Rawand A. Khasawneh, Sohaib B. Zoghoul, Yousef M. Henawi

**Affiliations:** 1 Department of Diagnostic Radiology and Nuclear Medicine, Faculty of Medicine, King Abdullah University Hospital, Jordan University of Science and Technology, Irbid, 22110 Jordan; 2 Orthopedic Division, Special Surgery Department, Faculty of Medicine, Jordan University of Science and Technology, Irbid, 22110 Jordan; 3 Department of Clinical Pharmacy, Faculty of Pharmacy, Jordan University of Science and Technology, Irbid, 22110 Jordan; 4 Radiology Department, Hamad Medical Corporation (HMC), Doha, Qatar 00000

**Keywords:** Hydatid cyst, Pediatric, Intramuscular, Paraspinal muscle, Thigh hydatid cyst, Case report

## Abstract

**Background:**

Hydatid disease is an endemic disease in many countries of the world including the Middle East. It mainly affects the liver and lungs. Intramuscular hydatid disease is rarely reported in children. Such uncommon localization of hydatid cyst may pose difficulties in the clinical and radiological diagnosis; hence affecting patient’s management and outcome even in endemic areas.

**Case presentation:**

We herein describe intramuscular hydatid cysts in 2 different children. The first case is a 5-year-old boy who presented with a painless palpable lump over the right lumbar paraspinal region. His history was remarkable for sheep contact. His laboratory results revealed a mild increase in white blood cell (WBC) count and C-reactive protein. The lesion showed typical features of a hydatid cyst on ultrasound. Further imaging including ultrasound of the abdomen and CT of the chest, abdomen, and pelvis showed infestation of the liver and lung as well. The lesions were resected surgically without complications. The patient received Albendazole preoperatively and after surgery for 3 months. No evidence of recurrence was seen during follow-up.

The second case is a 6-year-old girl who presented with an incidental palpable lump in her left thigh during her hospital admission for recurrent meningitis. Ultrasound and MRI imaging were performed demonstrating a unilocular cystic lesion in the left proximal rectus femoris muscle. A provisional diagnosis of hematoma vs. myxoma was given. Biopsy was performed and yielded blood products only. The lesion was resected surgically with a postoperative diagnosis of hydatid cyst. Blood tests performed afterward showed a positive titer for Echinococcus. The patient received Albendazole for 3 months. No evidence of recurrence was seen during follow-up.

**Conclusions:**

Despite its rarity; skeletal muscle hydatid cyst should always be considered in the differential diagnosis of cystic muscle lesions in children in endemic areas even if imaging studies did not show any of the typical signs. This will improve patient outcome by preventing unnecessary cystic puncture which might lead to serious complications, such as anaphylaxis and local dissemination.

**Supplementary Information:**

The online version contains supplementary material available at 10.1186/s12887-021-02843-5.

## Background

*Echinococcus Granulosus* is a well-known tapeworm causing hydatid disease which is an endemic and a common public health problem in many countries of the Middle East, Mediterranean region, Africa, Asia, South America, and Australia [1–8]. Liver and lung hydatid disease constitute the majority in affected patients [1, 3–7, 9–17]. Still, hydatid disease can occur rarely in other viscera and much rarer in skeletal muscles [2, 3, 6]. Intramuscular infestation can pose difficulties in the diagnosis and the management as it lacks the typical clinical appearance especially if isolated [7]. Furthermore, intramuscular hydatid cyst can mimic abscess, hematoma, lymphatic malformation, synovial cyst as well as necrotic malignant soft tissue mass lesions [18–20]. Scarce studies have reported intramuscular hydatid cyst in children [7, 8, 15, 18, 21–24]. Herein we report rare localizations of hydatid cyst disease in the paraspinal and proximal thigh muscles in two different children; one of whom presented with an isolated primary intramuscular hydatid cyst.

The case reports in this study are presented in line with the CARE criteria [25].

## Case (1) presentation

A 5-year-old male presented to the pediatric surgery clinic with a slowly growing painless lump in the right side of the lumbar region over 6-months. The lump was bothering him while walking and sitting. No history of trauma at the site of the lump was recalled. The systemic review was negative for any other complaints. On clinical examination, the mass was firm with no elicitable tenderness. No overlying skin changes were noticed.

**Fig. 1:**
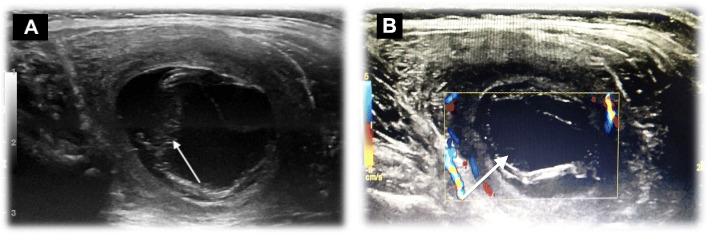
Ultrasound exam of the right Paraspinal region. **a**: A well-defined thick encapsulated cystic lesion is seen in the right paraspinal muscle measuring about 2.4 × 1.7 cm with water Lilly sign (arrow) consistent with hydatid cyst, **b** Doppler ultrasound of the same lesion showing hyperemia of the capsule and the daughter cysts (arrow)

**Fig. 2:**
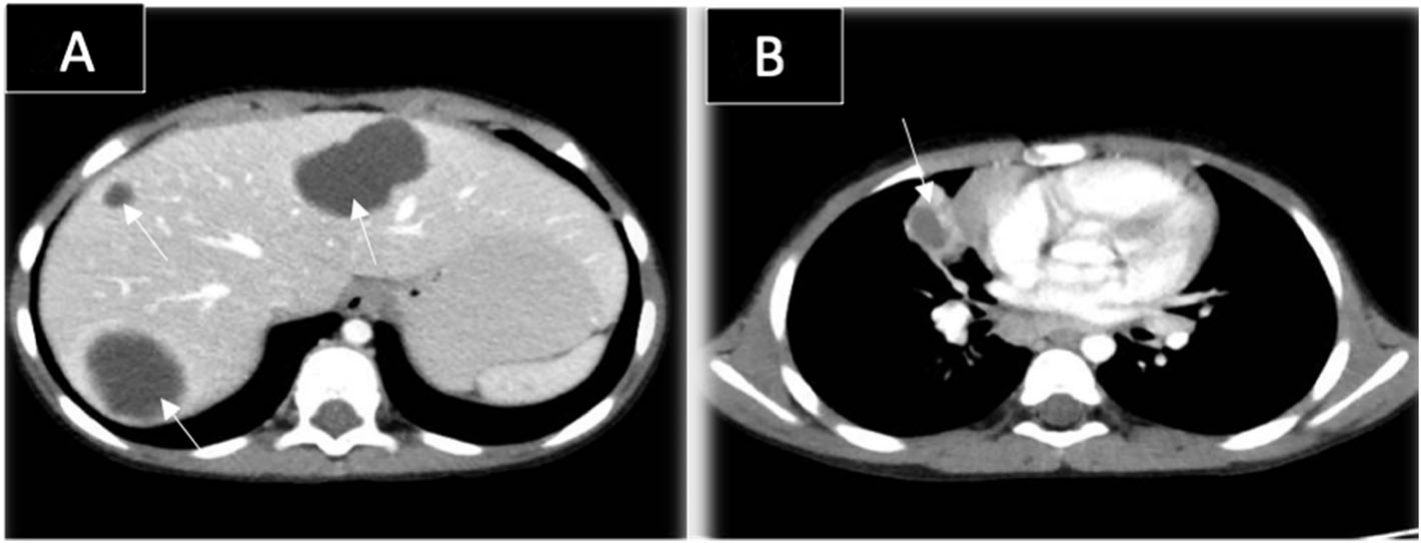
Enhanced axial CT scan of the chest and abdomen. **a**: showing multiple liver cysts (arrows). **b**: showing a Multi-loculated cystic lesion in the medial segment of the right middle lung lobe with a surrounding area of collapse consolidation (arrow); representing complicated hydatid cyst

**Fig. 3:**
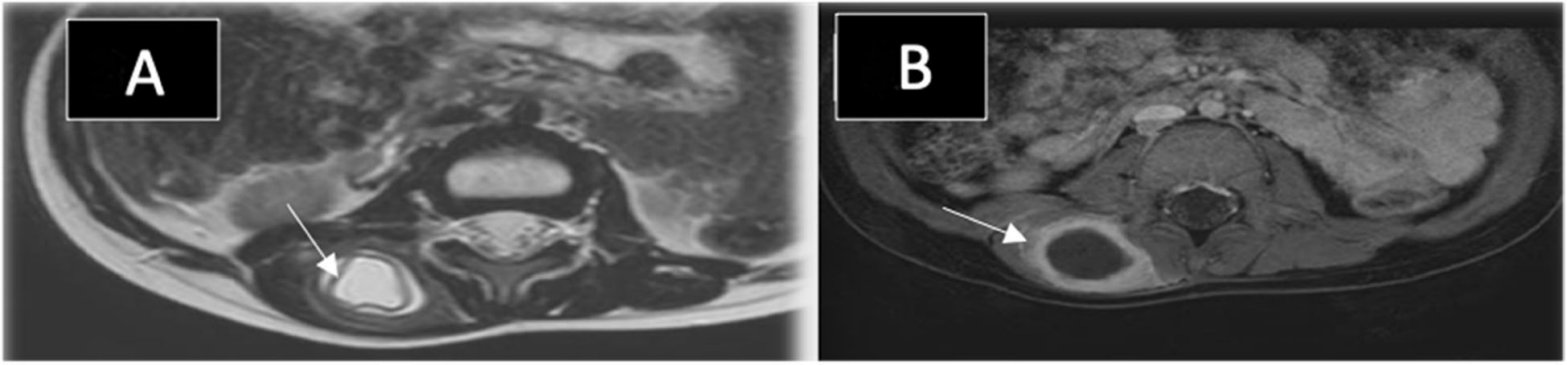
MRI of the lumbar spine in the axial planes. **a**: Axial T2 weighted images demonstrating the right Paraspinal hydatid cyst with water Lilly sign (arrow). **b**: Axial T1 postcontrast MRI with fat sat showing thick enhancement of the cyst capsule (arrow)

His blood tests were all normal except for a mild increase in white blood cell count (WBC) (12 × 10*3/mm*3) and C-reactive protein (48 mg/L). Ultrasound exam showed a well-defined thick encapsulated cystic mass lesion in the right paraspinal muscle measuring about 2.4 × 1.7 cm. Water Lilly sign membrane was seen within it (Fig. 1a). Multiple small cysts (daughter cysts) and hyperemia of its capsule on Doppler images were also demonstrated (Fig. 1b). A provisional diagnosis of intramuscular hydatid cyst was made. Further history revealed that the patient had close contact with sheep. Abdominal ultrasound showed multiple hydatid cysts in the liver. Computed Tomography (CT) scan of the chest, abdomen, and pelvis re-demonstrated the liver (Fig. 2a) and right paraspinal muscle hydatid cysts. In addition, a well-defined multi-loculated cystic lesion was seen at the medial segment of the right middle lung lobe with an adjacent area of collapse consolidation, representing a complicated hydatid cyst (Fig. 2b). Enhanced lumbar spine Magnetic Resonance Imaging (MRI), was ordered for preoperative planning. It showed the right paraspinal hydatid cyst as a cystic lesion spanning the levels of L2 to L3 vertebrae with a peripheral thick enhancing capsule, containing Water Lilly sign and daughter cysts. There was a reactive enhancement of the involved muscles as well (Fig. 3a, b). No deeper extension of the cyst was seen.

Upon radiological diagnosis, the patient was started on Albendazole 170 mg for 1 week before surgery. Blood serology showed a negative antibody titer for the *Echinococcus Granulosus* (8.1) En-bloc surgical excision of the paraspinal cyst was performed. The liver and lung hydatid cysts were subsequently excised in two separate procedures through open laparotomy and thoracotomy. R vacuum drain was placed postoperatively for 3 days after each procedure. The patient received Albendazole (170 mg) for 3 months postoperatively.

Histopathology confirmed the diagnosis of hydatid cyst disease. No recurrence was observed in the 16 months follow-up period.

## Case (2) presentation

A 6-year-old female presented to the pediatric emergency room with a decrease in the level of consciousness, inability to walk, and fever. The past medical history was remarkable for documented recurrent meningitis secondary to dental problems. Physical examination showed positive meningeal signs. Her lab results including lumbar puncture showed a new attack of acute meningitis. Enhanced brain MRI was performed and was unremarkable. During her physical exam, a non-tender incidental lump was noted in the anterior aspect of her left proximal thigh. The mass was soft measuring about 5 cm. The patient did not recall any trauma to the affected region. No discoloration of the overlying skin or palpable lymphadenopathy was noted.

**Fig. 4:**
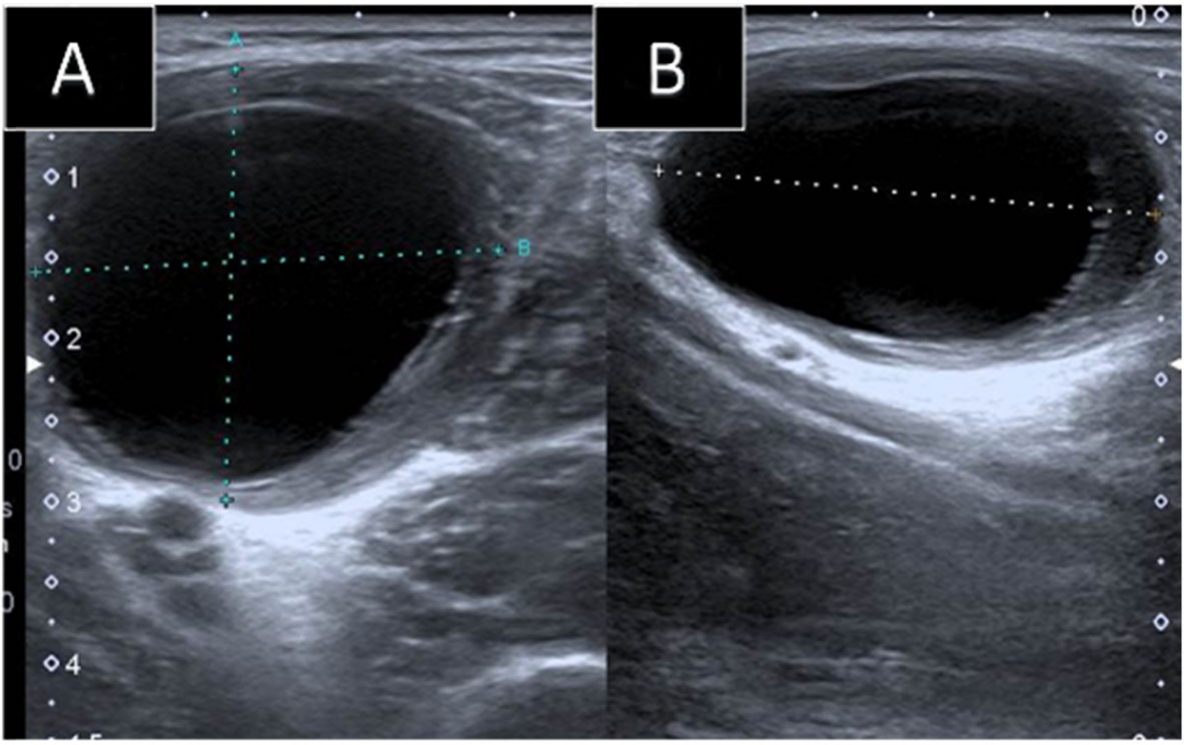
Ultrasound images of the left thigh. **a**: Trans, **b**: longitudinal: Showing a well-defined homogenous cystic lesion in the left rectus femoris muscle with posterior acoustic enhancement seen

**Fig. 5:**
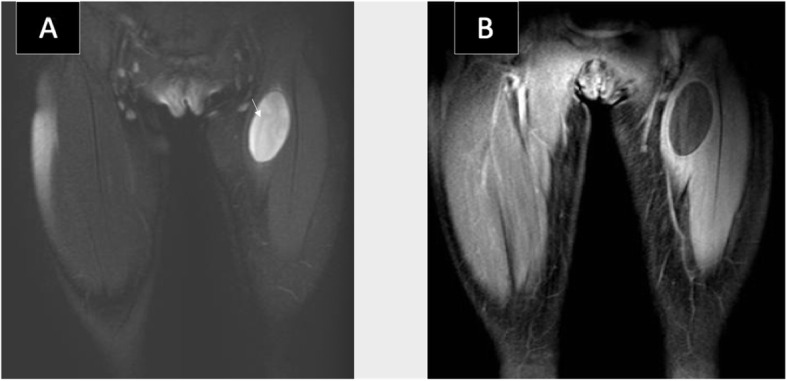
MRI of the thighs in coronal planes. **a**: STIR MRI image showing a well-defined cystic lesion with faint water Lilly sign (arrow) seen in the left rectus femoris muscle. **b**: T1 postcontrast image with fat saturation showing no perceptible enhancement of the lesion

Ultrasound showed a well-defined cystic lesion in the left rectus femoris muscle, with acoustic enhancement measuring about 2.7 × 5.2 × 2.4 cm. The cyst abutted the superficial femoral vessels. No internal vascularity, surrounding hyperemia, or soft tissue component was seen (Fig. 4a, b). The rest of the rectus femoris muscle fibers appeared edematous. A provisional diagnosis of chronic hematoma was given. Follow-up ultrasound after a month showed the same findings with no evidence of involution. Hence, an enhanced MRI of the left thigh was recommended to exclude other serious conditions. MRI revealed a well-defined oval-shaped intramuscular homogenous cystic lesion with no perceptible wall enhancement. (Fig. 5a, b). The given differential diagnosis for this incidental isolated intramuscular cyst was chronic hematoma versus intramuscular myxoma, despite its rarity in the pediatric age group.

A biopsy was advised. Aspirate of the cyst was performed and yielded blood cells only. The orthopedic surgeon decided to proceed with wide local excision. The mass was excised through an anterior proximal thigh incision overlying the mass. After isolating the femoral vessels, the mass was dissected out of the surrounding tissues and excised without rupture.

Surprisingly, histopathology came back as an intramuscular hydatid cyst. No history of animal contact was found upon further questioning. In a retrospective review of the MRI images, a faint water Lilly sign membrane on STIR images was recognized (Fig. 5a). Further laboratory exams showed a positive antibody titer for the *Echinococcus Granulosus* (30.1). CT scan of the chest, abdomen, and pelvis did not reveal any other organ infestation. The patient was started on oral Albendazole (170) mg for 3 months. No recurrence was noted during the 2 years follow-up period.

## Discussion and conclusions

Hydatid disease of the musculoskeletal system is rare with a reported incidence of about 1–5% [3, 5, 11, 12]. Primary intramuscular hydatid disease without liver and lung involvement is even rarer with an incidence of about 2–3% [4, 10, 14]. Few cases of lower limb isolated intramuscular hydatid cysts were reported in the literature in the pediatric age group (Table 1). None of the reported cases, however, were in the left rectus femoris muscle as seen in this study (case (2) presentation) (Table 1). Paraspinal muscles are much more rarely affected in hydatid disease [14]. Herein, we also report an unusual localization of hydatid cyst in the right paraspinal muscle, which was not reported before in pediatric patients (Fig. 1a, b and Fig. 3a, b). (Table 1). The reason behind the rare occurrence of hydatid cyst in skeletal muscles is their frequent contractility and high lactic acid content [1, 3–5, 7, 9, 10, 13–16, 19].

**Table 1:** Literature review of pediatric intramuscular hydatid cysts

Author	Age•	Sex	Location	Presenting symptom	Isolated vs. Multiple	Uniloculated vs. Multiloculated.
**Tekin et al** [7]	10	Female	Thigh M	Painless mass	Isolated	NA
	6	Female	Thigh M	Painless mass	Isolated	NA
**Ghoroobi et al** [15]	5	Male	Left Posterior distal thigh	Painless swelling	Isolated	Uniloculated
**Atalar et al** [8]	4	Female	Left Vastus Medialis M	Painless mass	Isolated	Uniloculated
**Erol et al** [21]	11	Male	Right Medical Gastroc. M	Painless mass	Isolated	Uniloculated
**Dudkiewez et al** [22]	14	Male	Right Vastus Medialis M	Painless mass	Multiple	NA
**Duygulu et al** [20]	8	Female	Left Sartorius M	Painless mass	Isolated	Multiloculated
**Landolsi et al** [24]	8	Male	Left semitendinosus M	Painless mass	Isolated	Multiloculated
**Kerimoglu et al** [18]	8	Female	Left flexor halluces longus M	Painful swelling	Isolated	Multiloculated
**Cankorkmaz et al** [23]	4	Female	Between left adductor M and iliopsoas M	Painless mass	Isolated	Uniloculated

*M* Muscle, *Gastroc.* Gastrocnemius, *NA* not available

•: Years

The average age for most of the cases reported in the literature was adult to middle age groups [1]. Scarce cases of intramuscular hydatid cyst were reported in children (Table 1). Both of the cases presented here were seen in the pediatric age group, adding to the challenge in diagnosis and treatment.

Intramuscular hydatid disease is frequently asymptomatic [15]. Painless slow-growing mass with normal overlying skin is the most commonly reported complaint in the literature [7, 10, 12, 13]. This is similar to the clinical presentation of the patients presented in this report.

Imaging plays a major role in diagnosing intramuscular hydatid cysts when typical imaging features are present. Ultrasonography is a major non-invasive tool to confirm the diagnosis of hydatid disease [7]. Typical ultrasound imaging features include the pathognomonic daughter cysts [16]. Double line sign is another characteristic sign in Ultrasound of hydatid cysts [7].

The CT appearance of the hydatid cyst is variable [5]. Hydatid cyst on CT may mimic tumor if presented as a solid mass secondary to inflammatory changes [5]. However, the presence of daughter cysts, germinal epithelium detachment, and wall calcifications may confirm the diagnosis [7]. CT is also superior for the assessment of bone invasion by the cysts [7].

MRI has a major diagnostic role in assessing the extent of skeletal muscle infestation, exclude other possible etiologies, and for surgical planning [1, 10, 14, 16]. MRI can reveal a cystic mass containing daughter cysts, rim sign, and Water Lilly sign [6, 17]. The rim sign is considered a characteristic sign in muscular hydatidosis [5, 8]. It is defined as a low signal intensity rim seen around the cyst, likely formed by the peri-cyst, most evident on T2 weighted images (WI’s).

The diagnosis of hydatid cyst was made easily by imaging in the first case despite its atypical clinical presentation. Daughter cysts and Water Lilly sign were seen in all imaging modalities. The diagnosis was also supported by the patient’s positive history of sheep contact and coincident liver and lung cysts upon further imaging.

The diagnosis in the second case, however, was more challenging. The possibility of an intramuscular hydatid cyst was not raised neither clinically nor radiologically. Biopsy was also performed and was not diagnostic. The final diagnosis came out only after surgical resection and histopathological analysis. Its ultrasound imaging appearance as a unilocular mostly homogenous cystic lesion (Fig. 4a, b) may reflect on the initial stage of parasitic infestation in muscles [5].

Serological tests, like the indirect hemagglutination assay test (IHA), can be valuable in making the diagnosis when they are positive [16]. Unfortunately half of the primary skeletal muscle hydatidosis gives false -negative results in serology [8, 14, 16, 19]. The serology in the first presented case was negative despite multi-organ infestation, while it was positive in the second case although it was an isolated intramuscular hydatid cyst.

There are multiple treatment options for hydatid cyst. En bloc surgical resection is considered the treatment of choice with lower complications and recurrence rates [2, 4, 14]. Other Options include PAIR (percutaneous aspiration injection-re-aspiration); which showed promising results lately; medical treatment (Albendazole), and watch and wait [2]. Both patients in this series were treated with en bloc surgical resection along with Albendazole before and after surgery in the first case, while only after surgery in the second case, as the diagnosis was revealed only after surgery.

In conclusion; pediatric intramuscular hydatid cyst is rare. A painless lump is the usual clinical presentation. Radiology plays a crucial role in making the diagnosis of hydatid cyst when typical imaging features are seen. Surgery, and chemotherapy are the standard treatment options. Atypical clinical and radiological findings may result in mismanagement of the patient leading to serious complications. Intramuscular hydatid cyst should be kept in the differential diagnosis for any cystic muscular lesion in endemic areas.

## Supplementary Information

**Additional file 1.** Timeline for case 1.

**Additional file 2.** Timeline for case 2.

## Data Availability

All data generated or analyzed during this study are included in this manuscript and its supplementary information files.

## Abbreviations

WBC: White Blood Cell
CT: Computed Tomography
MRI: Magnetic Resonance Imaging
STIR: Short Tau Inversion Recovery
WI’s: Weighted images
IHA: Indirect Hemagglutination Assay
PAIR: Percutaneous Aspiration Injection Re-aspiration
