# Supplementary material for: Unusual intramuscular locations as a first presentation of hydatid cyst disease in children: a report of two cases
Source: BMC Pediatr. 2021 Aug 31;21:371. doi: 10.1186/s12887-021-02843-5 (PMC8406844; doi:10.1186/s12887-021-02843-5)
Supplement: Supplementary file 1 — Additional file 1. Timeline for case 1. [file 12887_2021_2843_MOESM1_ESM.docx]

Presented to the pediatric surgery clinic with a painless lump in his right paraspinal region that was bothering him while sitting or walking. No tenderness or skin changes were seen over the lesion

A previously healthy 5-year-old male

9/2018

2/2020

Follow up at the pediatric infectious disease and pediatric surgery clinics with no clinical evidence of disease recurrence.

4/2019

Follow up ultrasound and CT scan of the chest , abdomen and pelvis with no radiological evidence of recurrence

10/2018

Blood tests showed elevated White blood cell count and c- reactive protein

The patient was discharged on Albendazole 170mg for 3 months

170mg for 3 months

Surgical resection of the lesions by pediatric surgeon in 2 different surgical sessions with uneventful postoperative course

The patient was started on Albendazole

170mg

MRI of the lumbar spine was performed with a provisional diagnosis of for preoperative planning of the right paraspinal cyst which showed no deeper extension of the lesion

CT scan of the chest, abdomen and pelvis was ordered that showed the above lesions in addition to right middle lung lobe hydatid cyst

Ultrasound was performed to characterize the lump with sonographic findings diagnostic of hydatid cyst with evidence of liver infestation as well

Last follow up visit ; the patient is doing well with no clinical or radiological evidence of disease recurrence
