# Supplementary material for: Unusual intramuscular locations as a first presentation of hydatid cyst disease in children: a report of two cases
Source: BMC Pediatr. 2021 Aug 31;21:371. doi: 10.1186/s12887-021-02843-5 (PMC8406844; doi:10.1186/s12887-021-02843-5)
Supplement: Supplementary file 2 — Additional file 2. Timeline for case 2. [file 12887_2021_2843_MOESM2_ESM.docx]

6-year-old female with history of recurrent meningitis

9/2017

Incidental lump in her left upper thigh during her hospital admission for meningitis. No tenderness or skin changes were seen over the lesion

2/2020

Last follow up ; the patient is doing well with no clinical or radiological evidence of disease recurrence

Follow up ultrasound of the left thigh with no evidence of recurrence

Follow up at the pediatric infectious disease and orthopedic clinics with no new complaints regarding the thigh lesion

6/2018

12/2017

Ultrasound was performed to characterize the lump with a provisional diagnosis of hematoma

Blood tests performed showed positive titer for Echinococcus

CT of the chest and abdomen and pelvis was performed with no evidence of other organs infestation

The patient was started on Albendazole

170mg for 3 months

Histopathology report came out as hydatid cyst

Surgical resection of the lesion by orthopedic surgeon

2/2018

Biopsy of the lesion which yielded only blood

1/2018

MRI of the thigh was performed with a provisional diagnosis of intramuscular hematoma or myxoma

Follow up ultrasound peformed with no change in the size or shape of the lesion

11/2017
